# Comparison of Methods of Detecting IL-1β in the Blood of Alzheimer’s Disease Subjects

**DOI:** 10.3390/ijms26020831

**Published:** 2025-01-20

**Authors:** Alexandra D. Remnitz, Roey Hadad, Robert W. Keane, W. Dalton Dietrich, Juan Pablo de Rivero Vaccari

**Affiliations:** 1 Department of Neurological Surgery and the Miami Project to Cure Paralysis, University of Miami Miller School of Medicine, Miami, FL 33136, USA; aremnitz@med.miami.edu (A.D.R.); rkeane@miami.edu (R.W.K.); ddietrich@med.miami.edu (W.D.D.); 2 Department of Physiology and Biophysics, University of Miami Miller School of Medicine, Miami, FL 33136, USA; rhadad@miami.edu

**Keywords:** biomarkers, inflammasome, interleukin, cytokines, IL-1 β, Alzheimer’s disease

## Abstract

Interleukin (IL)-1β is a pro-inflammatory cytokine whose levels are increased in the brains of Alzheimer’s disease (AD) patients. Despite the role of IL-1β in the pathology of AD, the fact that it is expressed at very low levels makes it a challenging cytokine to measure, hence limiting its potential use as a reliable biomarker. Moreover, being able to accurately and reliably measure the levels of IL-1 β in blood makes it possible to evaluate this cytokine as a potential biomarker of the inflammatory response in AD. In this study, we compared three quantification methodologies, Meso-Scale Discovery (MSD), both V-Plex and S-Plex versions, and Quanterix’s SIMOA (Single-Molecule Array), to measure IL-1β in the serum of AD patients and age-matched controls. These assays are routinely used to measure IL-1β serum levels with high specificity and sensitivity in human AD patients, yet to the best of our knowledge, no study has compared all three techniques for their accuracy to measure IL-1β as biomarkers. Our findings indicate the two MSD assays can be used to measure IL-1β levels in AD and control serum, but the SIMOA assay showed the highest receiver operating characteristics (ROCs), with an area under the curve (AUC) of 0.9532, which can be compared to the AUC values for the V-Plex assay, 0.5660, and the S-Plex assay, 0.6632. Taken together, these data show that although all technologies are useful in the measurement of IL-1β in the blood, the SIMOA IL-1β 3.0 assay is more reliable and sensitive in measuring biomarkers of AD.

## 1. Introduction

Interleukin (IL)-1β is a key cytokine involved in the inflammatory response in a variety of diseases, and it has been linked to Alzheimer’s disease (AD) pathology [1,2,3,4,5,6,7,8]. Inflammasomes are multiprotein complexes that play a key role in the innate immune system by detecting pathogenic microorganisms and stressors and activating inflammatory responses that lead to the production of IL-1β [9,10]. The NLRP3 inflammasome is the most studied inflammasome in AD pathogenesis. It consists of the sensor protein NLRP3 (NOD-like receptor family pyrin domain containing 3), the adaptor protein ASC (apoptosis-associated speck-like protein containing a caspase recruitment domain), and the effector enzyme pro-caspase-1 [11]. The activation of the inflammasome leads to the cleavage of pro-caspase-1 into its active form, caspase-1. Active caspase-1 then processes the inactive precursor of IL-1β (pro-IL-1β) into its active form, IL-1β. IL-1β is then secreted from the cell to exert its pro-inflammatory effects [11].

In AD, the accumulation of amyloid-β (Aβ) plaques and neurofibrillary tangles (NFTs) are characteristic pathological features [12]. AD is the most common type of dementia, resulting in about 60 to 80% of all cases, and it is characterized by difficulty in remembering recent social interactions and events as well as depression. Moreover, AD patients also present difficulties with behavior such as poor judgement and issues with communication, and in the later stages of this disease, AD patients may present difficulty walking, speaking, and swallowing [13], all of which are associated with the accumulation of Aβ plaques outside neurons and tau inside neurons, leading to neuronal cell death, inflammation, and brain atrophy [14,15,16,17,18].

Aβ plaques can activate the NLRP3 inflammasome in microglia, the resident immune cells of the central nervous system (CNS). Once activated, the NLRP3 inflammasome promotes the production and release of IL-1β, leading to chronic neuroinflammation [19]. Chronic neuroinflammation, driven by IL-1β and other cytokines, contributes to neuronal damage and the progression of AD. Elevated levels of IL-1β have been observed in the brains of patients with AD, further supporting its role in AD pathology [20]. Thus, targeting the inflammasome or IL-1β signaling pathways presents a potential therapeutic strategy for reducing neuroinflammation in AD. Accordingly, inhibitors of the inflammasome and IL-1β are being investigated for the treatment of AD [21].

IL-1β has been shown to induce the proliferation of neuroinflammatory cells, such as astrocytes and microglia, as well as macrophages [22]. Since the inflammasome is able to mediate the release of IL-1β and IL-18, which can trigger a cascade of secondary inflammatory events in neuroinflammation, this multiprotein complex is a critical mediator of neuroinflammation and a potential therapeutic target in the treatment of several neurodegenerative diseases. In AD, the activation of the inflammasome by Aβ plaques leads to increased levels of IL-1β, contributing to chronic neuroinflammation and disease progression [23].

Despite the importance of IL-1β in a variety of diseases, the fact that it is present at very low levels in the blood of humans makes it a very challenging analyte to measure as a reliable biomarker of the inflammatory response. Fortunately, new technologies have been developed to more accurately and sensitively measure the levels of this well-known pro-inflammatory cytokine. One of these technologies involves the use of single-molecule array (Simoa) technology to quantify IL-1β, and in the other, IL-1β is quantified using enhanced chemiluminescent immunoassay (ECLIA) technology. Thus, being able to properly measure IL-1β levels is crucial to overcome the challenges associated with IL-1β being present at very low levels in the blood, which makes it extremely challenging to measure this cytokine in humans. Therefore, in this study, we aimed to compare methods of detecting IL-1β using sensitive assays in order to find the most effective detection methods for this critical cytokine.

## 2. Results

### 2.1. IL-1β Levels in Serum Are Higher in Patients with AD When Compared to Those in Healthy Controls

**Figure 1:**
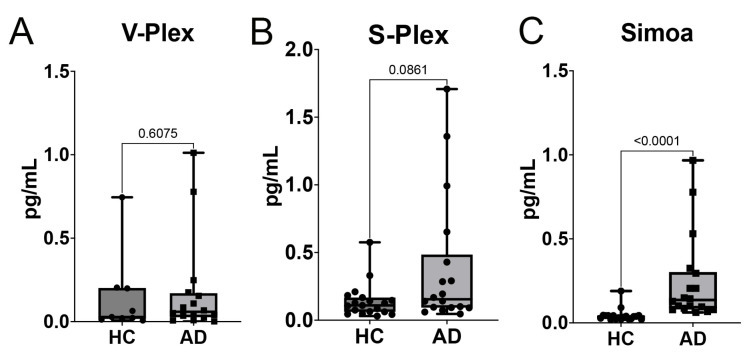
Comparison of IL-1β biomarker levels between healthy controls (HCs) and Alzheimer’s disease (AD) patients across different assays. Protein levels are given in pg/mL for IL-1β measured with the (**A**) V-Plex, (**B**) S-Plex, and (**C**) Simoa assays for healthy controls (HCs) and AD subjects. V-Plex: N = 9 HCs and 16 AD patients; S-Plex: N = 19 HCs and 18 AD patients, Simoa: N = 19 HCs and 18 AD patients. All assays were carried out with the same number of samples (19 HCs and 18 AD patients), but the difference in the sample size between groups is due to the sample values being out of the range of the respective assays. Each dot represents an individual sample, with boxes indicating the minimum and the maximum values. Statistical significance between groups is indicated above each comparison.

IL-1β levels have been shown to be elevated in patients with AD [24]; however, IL-1β is well known to be a difficult cytokine to measure due to its low abundance in blood. To compare three different detection methods in order to determine the most optimal way of measuring the levels of IL-1β in healthy controls (HC) and AD patients, we used V-PLEX (Figure 1A), S-Plex (Figure 1B), and Simoa (Figure 1C) technologies. Our data indicate that the protein levels of IL-1β in the serum were higher in AD patients than in the controls; however, the most significant data were obtained only with Simoa. Accordingly, with Simoa, when comparing HC vs. AD, IL-1β levels presented a mean of 0.04 for HC and 0.24 for AD (range: HC = 0.02–0.19, AD = 0.06–0.97). However, there was no significant difference when comparing the same samples using V-Plex (range: HC = 0.007–0.75, AD = 0.001–1.01) and S-Plex (range: HC = 0.03–0.58, AD = 0.05–1.71). Together, these findings indicate that Simoa is more sensitive in discerning between HC and AD patients.

### 2.2. ROC Analysis of IL-1β

**Figure 2:**
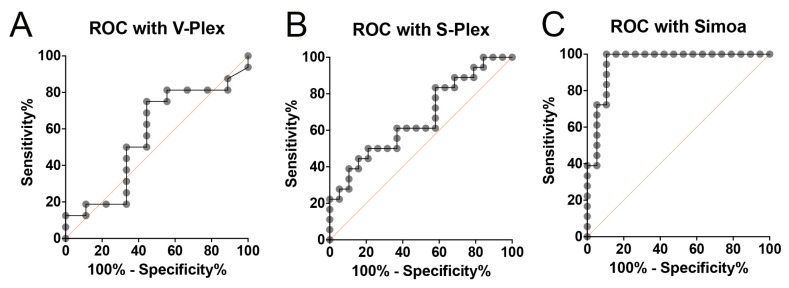
Receiver operating characteristic (ROC) curves for discriminating AD from HC using IL-1β biomarkers. ROC curves are shown for (**A**) V-Plex, (**B**) S-Plex, and (**C**) Simoa assays. The orange diagonal line represents the line of no discrimination (AUC = 0.5). V-Plex: N = 9 HCs and 16 AD patients; S-Plex: N = 19 HCs and 18 AD patients; Simoa: N = 19 HCs and 18 AD patients. All assays were carried out with the same number of samples (19 HCs and 18 AD patients), but the difference in the sample size between groups is due to the sample values being out of the range of the respective assays.

To compare the three methods of IL-1β detection in terms of sensitivity and specificity for biomarker detection, we determined the area under the curve (AUC) to obtain the receiver operating characteristics (ROCs) between the V-plex, S-plex, and Simoa assays. ROC analysis is used to quantify how accurately medical diagnostic tests can discriminate between two patient states, in this case, HC and AD patients. When comparing the three methods of IL-1β detection utilizing ROC, Simoa presented the greatest AUC, indicating that the Simoa IL-1β 3.0 assay is a more sensitive biomarker platform for measuring IL-1β in blood samples (Figure 2). Accordingly, the AUC for Simoa was 0.95, which can be compared to 0.57 for V-Plex and 0.67 for S-Plex (Table 1). Moreover, with a cut-off point of 0.05 pg/mL, the Simoa assay presented 100% sensitivity with 89% specificity, and the S-Plex assay showed 83% sensitivity and 42% specificity with a cut-off point of 0.09 pg/mL. Furthermore, the cut-off point for V-Plex was 0.03 pg/mL, with 75% sensitivity and 56% specificity (Table 2). Together, these findings indicate that Simoa offers greater potential for discriminating between unaffected (HC) and AD subjects than the other two technologies, results that are consistent with Simoa being the most sensitive technology of the three used in the present study. However, all three assays had similar cut-off points in the range of 0.03 to 0.09.

### 2.3. Comparison Within Groups and Between Assays

**Figure 3:**
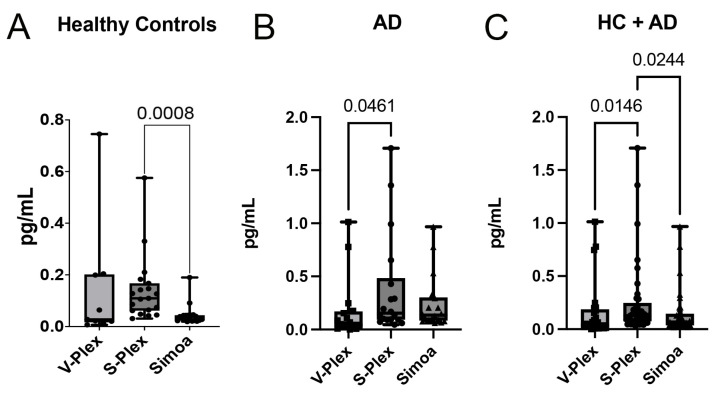
Comparison of IL-1β biomarker levels for different cohorts (HCs, AD subjects, and combined group). Protein levels are given in pg/mL for IL-1β measured with the V-Plex, S-Plex, and Simoa assays for (**A**) HCs, (**B**) AD subjects, and (**C**) HC+AD. (**A**) HC= V-Plex: N = 9; S-Plex: N = 19; Simoa: N = 19. (**B**) AD = V-Plex: N = 16; S-Plex: N = 18; Simoa: N = 18. (**C**) HC+AD = V-Plex: N = 25; S-Plex: N = 37; Simoa: N = 37. Each dot represents an individual sample, with boxes indicating the minimum and maximum values. Statistical significance between groups is indicated above each comparison.

We then aimed to determine the effectiveness of the three methods within groups and between assays. Thus, we compared the levels of IL-1β in the HC (Figure 3A) and AD samples (Figure 3B) as well as the full cohort of subjects in this study, namely, HC+AD (Figure 3C). When comparing the three methods of IL-1β detection within groups and between assays, we found that Simoa presented greater detection ability within the lower range of IL-1β measurement for the HCs (Figure 3A); however, the levels were very similar for all three methods in the AD cohort (Figure 3B), and in the full cohort, S-Plex presented higher levels of IL-1 β than the other two groups (Figure 3C).

**Figure 4:**
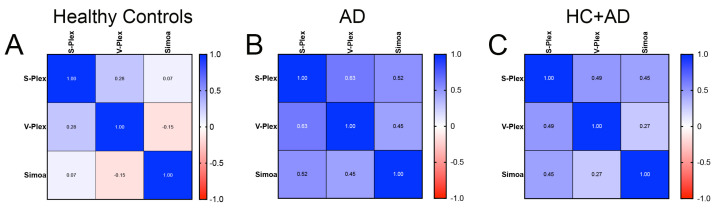
Correlation matrices comparing IL-1β protein levels across different assays. Panels represent correlation analyses for (**A**) HCs, (**B**) AD subjects, and (**C**) the combined group (HC + AD) between S-Plex, V-Plex, and Simoa assays. Color intensity and values within cells indicate the strength and direction of correlations (blue: positive correlation; red: negative correlation).

Then, we carried out correlation matrix analyses to study the relationship between IL-1β levels obtained from the V-Plex, S-Plex, and Simoa assays (Figure 4). Our findings indicate a high correlation between the three assays in the AD group (Figure 4B) but a low correlation between the three assays in the HC group (Figure 4A). Taken together, these results indicate that MSD and Simoa are effective assays for measuring IL-1β when the values are high, such as those affected by disease, in this case, AD. However, the Simoa assay was more optimal when measuring very low levels of IL-1β.

### 2.4. Differences Between Assays

**Figure 5:**
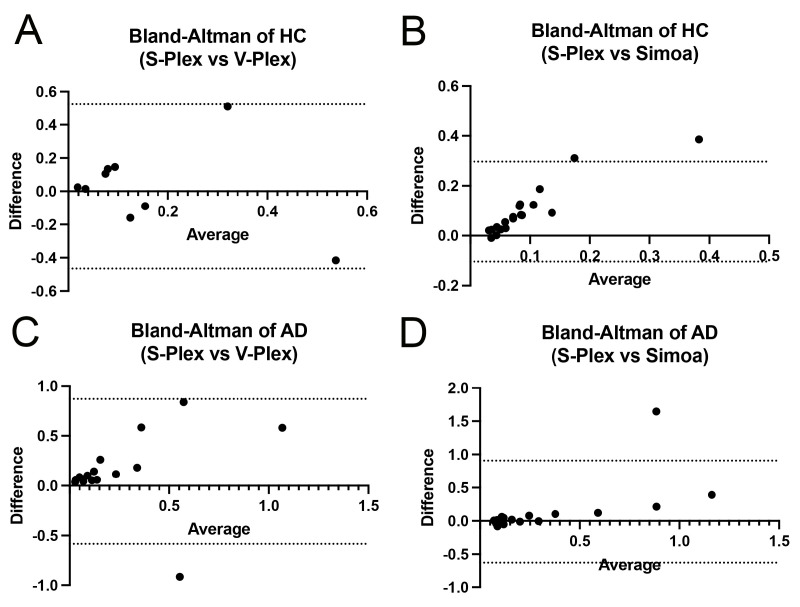
Bland Altman analysis between assays. Bland–Altman plot of the agreement between assays for (**A**) S-Plex and V-Plex; (**B**) S-Plex and Simoa in the HC cohort; (**C**) S-Plex and V-Plex and (**D**) S-Plex and Simoa in the AD cohort.

Finally, to further determine the differences between the assays, we performed Bland–Altman plot analyses between the S-Plex and V-Plex (Figure 5A) assays and the S-Plex and Simoa assays (Figure 5B) in the HC cohort as well as between the S-Plex and V-Plex assays (Figure 5C) and the S-Plex and Simoa assays in the AD cohort (Figure 5D). The Bland–Altman plot is used to plot the differences between assay measurements against their averages in order to visualize the agreement between two methods. When comparing the agreement between S-Plex and V-Plex after measuring the samples in the HC group (Figure 5A), we found a trend of heteroskedasticity, as determined by noting a funnel-like pattern, and the bias was 0.03. Moreover, there was high agreement between the lower values. However, there was some disagreement among the mid-range and higher values, and the 95% limit of agreement was between −0.5 and 0.5.

When comparing the agreement between S-Plex and Simoa after measuring the samples in the HC group (Figure 5B), the bias was determined to be 0.1, and there was an increase in the level of disagreement as the values started to increase, and the 95% limit of agreement was between −0.1 and 0.3.

When comparing the agreement between S-Plex and V-Plex after measuring samples in the AD group (Figure 5C), the bias was found to be 0.14, and there was also an increased level of disagreement between assays as the values increased in the mid and high ranges. Furthermore, the 95% limit of agreement was between −0.58 and 0.87.

When comparing the agreement between S-Plex and Simoa after measuring samples in the AD group (Figure 5D), the bias was found to be 0.14, and there was not much of an increase in the level of disagreement between assays across the measured values. Furthermore, the 95% limit of agreement was between −0.63 and 0.91.

Together, these findings indicate a high level of agreement between S-Plex and Simoa when measuring samples in the blood of AD patients when protein levels of IL-1β are higher than in the HCs. Furthermore, the bias in all cases was close to zero, suggesting that the assays produce similar results.

## 3. Discussion

In this study, three different methods of IL-1β detection were used: MSD’s V-plex and S-plex as well as the Simoa IL-1β 3.0 kit that was run using HD-X. We aimed to compare these detection methods to determine the most sensitive method for the detection of IL-1β in healthy and affected individuals with AD known to present an inflammatory response associated with the inflammasome [25]; thus, serum from AD patients was used as previously described [25].

The ECLIA was carried out using Meso-Scale Discovery (MSD) Immunoassay kits. The MSD V-Plex kit consists of a capture antibody attached to a working electrode that binds a potential biomarker, which is then enhanced by a SULFO-TAG-labeled detection antibody that allows for assays to be very sensitive in the detection of potential biomarkers. The plate electrodes are electrified using the MESO-Quickplex instrument, causing the SULFO-TAG labels to emit light. In addition, S-Plex, which is capable of measuring analytes at the fg/mL level, is an ultrasensitive assay kit that consists of a streptavidin/biotin antibody complex bound to a working electrode that recognizes a potential biomarker/analyte, which is then bound by a TURBO-BOOST antibody, which is enhanced using a TURBO-TAG. Simoa HD-X is an automated system that uses single-molecule array technology to detect proteins at very low levels, even at the fg/mL scale. Simoa technology uses capture antibodies bound to magnetic beads and detection antibodies that generate fluorescence, forming a bead/biomarker/detection antibody complex so that each bead is capable of binding one analyte when this analyte is available. These complexes are then loaded onto a disc, and then enzymatic signal amplification with a fluorescent substrate is carried out for further imaging and data processing.

In this study, the ROC analysis shows that the Simoa assay performed excellently in distinguishing IL-1β protein levels in the serum of AD patients from non-AD patients with a high AUC (0.95). V-Plex, in contrast, showed minimal sensitivity for discriminating IL-1β in AD and non-AD subjects based on an AUC of 0.57 when compared to S-Plex and Simoa. Moreover, Simoa outperformed the other two assays, with a sensitivity of 100% and a specificity of 89%, indicating that Simoa is highly effective in measuring IL-1β with high sensitivity. On the other hand, V-Plex and S-Plex showed moderate sensitivity but lower specificity and accuracy, indicating a higher likelihood of false positives or false negatives compared to the Simoa IL-1β 3.0 assay.

The Bland–Altman plots show the difference between the measurements of IL-1β from the two different assays compared to their average. This analysis helped identify whether the assays can be used interchangeably or if there are significant discrepancies. In this study, our data from the Bland–Altman plots together with the correlation matrix showed that for HCs, there were slight similarities between both MSD assays. However, these similarities were even greater in the AD samples. Similarly, for the AD samples, there was a great degree of similarity between the MSD and Simoa assays, indicating that when the concentrations of IL-1β are high, all three assays are similarly effective.

IL-1β is a central mediator of inflammation and the immune response, playing critical roles in defending the body against infections, initiating repair processes, and maintaining homeostasis [26]. Dysregulation of IL-1β can contribute to chronic inflammatory diseases, autoimmune disorders, and other pathological conditions. IL-1β has key roles in inflammatory responses, tissue repair, and cellular stress [27]. IL-1β is crucial for the activation and recruitment of immune cells, such as neutrophils and macrophages, to sites of infection or injury to help the body respond to tissue damage [28]. In addition, IL-1β is a potent pro-inflammatory cytokine that promotes the expression of adhesion molecules on endothelial cells, facilitating the migration of immune cells to an affected area [29]. Furthermore, IL-1β acts on the hypothalamus to induce fever, which is a common response to infection that helps inhibit the growth of pathogens [30]. IL-1β stimulates the production of other inflammatory cytokines, such as tumor necrosis factor (TNF) and IL-6, hence amplifying the inflammatory response [31]. Moreover, IL-1β promotes the differentiation of naive T cells into Th17 cells, a subset of T cells that plays a role in autoimmune diseases and defense against extracellular pathogens [32] and contributes to the activation and differentiation of B cells in the process of antibody production [33].

AD is the most common form of dementia, and its pathogenesis is characterized by the extracellular accumulation of Aβ plaques, which is a principal event that initiates cellular responses mediated by microglia [34,35] and can trigger inflammasome activation [36]. Furthermore, inflammasome inhibition has been shown to result in the protection of spatial memory loss and a decrease in Aβ deposition in AD mice [37,38]. In AD, the accumulation of Aβ in the CNS activates microglia, which results in the release of IL-1β [39]. Importantly, Aβ plaques form in part when Aβ monomers bind to ASC specks [40]. In addition, studies have demonstrated that the formation and accumulation of tau are associated with the secretion of inflammasome components from microglia [41]. Moreover, inflammasome proteins have been shown to be present in the brains of AD patients [42] as well as the blood of subjects in the early stages of cognitive changes [25,43]. Taken together, these findings suggest that the inflammasome contributes to the pathological mechanisms of AD. Thus, the disruption of inflammasome activation could be a potential target for AD therapy.

To date, the only biomarker of the inflammatory response associated with AD pathology that is well accepted in the field is glial fibrillary acidic protein (GFAP) [44]. However, GFAP is more a marker of astrogliosis than the broad inflammatory response that may precede or follow the onset of AD. In this vein, this study and others [25,42,43,45,46] suggest that the inflammasome is a promising biomarker of the inflammatory response in AD associated with a variety of cells since neurons [47], astrocytes [48], microglia [49], oligodendrocytes [50], and endothelial cells [51] express a variety of inflammasomes capable of releasing IL-1β. Thus, reliable measurements of IL-1β offer great potential for the monitoring of the inflammatory response in AD. However, the purpose of this study was to identify suitable methods for measuring IL-1β with sensitivity that is high enough to allow the results to be reliably applied to the monitoring of patients for research purposes, given the importance of the inflammasome to the pathology of AD and given the role that IL-1β plays in the inflammasome signaling pathway. The small sample size used in this study did not allow for the testing of IL-1β as a diagnostic biomarker of AD. Therefore, research is underway to study the diagnostic potential of IL-1β as a biomarker of the inflammatory response associated with cognitive changes.

Furthermore, the AUC detected for inflammasome biomarkers such as ASC [25,43] and IL-1β in this study, with values above 0.9, indicates that the inflammasome is on par with the AUC detected for pTau_217_ [52] and higher than for GFAP [53]. However, larger studies, beyond the present hypothesis-generating study, are needed to confirm whether the inflammasome is an early biomarker of the inflammatory response in AD pathology.

Taken together, we envision that a blood panel will eventually become available for patients, in which astrogliosis can be assessed by measuring the levels of GFAP, innate immune inflammation can be assessed with the blood levels of ASC and IL-1β, microglia status can be assessed with TREM2, neuronal status can be assessed with neurofilament light (NfL) and pTau_217_, and amyloidosis can be assessed with amyloid-β (Aβ42:40) levels. Analysis of a blood-based biomarker panel of this nature has the potential to dictate the development of specific therapies for treating these different biomarkers and eventually indicate which therapies are most suitable at different disease stages and under different molecular conditions.

Finally, another important point when considering different technologies is the cost associated with the use of a particular technology and the broad availability of such instruments in laboratories. For instance, in this case, the HD-X instrument, due to its sophistication, is a technology that comes with a higher cost when compared to the MESO-Quickplex instrument. Furthermore, when comparing cost per sample, the current estimates suggest that the Simoa IL-1β 3.0 kit entails approximately 1.7 higher costs than the S-Plex IL-1β kit per sample.

## 4. Materials and Methods

### 4.1. Study Design

We previously analyzed the cohort of patients used in this study to analyze how inflammasome-signaling proteins in serum serve as biomarkers of the inflammatory response in patients with mild cognitive impairment and AD [25]. In this study, samples from 19 unaffected controls and 18 AD samples from AD patients were analyzed with two different platforms: Simoa HD-X (Quanterix, Billerica, MA, USA) and MESO-QuickPlex (MSD, Rahway, NJ, USA). All samples were analyzed using both instruments, and 2 different types of kits were used for the MSD technology: S-Plex and V-Plex. Then, ROC and pertinent biomarker characteristics were calculated for each assay (Simoa, S-Plex and V-Plex), and correlations and Bland Altman analyses were performed.

### 4.2. Patients

The samples, obtained after informed consent was acquired, were purchased from BioIVT (Hicksville, NY, USA) under IRB number 20170439. Samples corresponded to 18 samples from AD patients (12 males and 6 females) and 19 controls (7 males and 12 females). The age range for the AD cohort was 47 to 84 years old (median age: 63), and that for the control cohort was 55 to 80 (median age: 60). Patients were classified according to their ARIC MRI cognitive function scores [54]. Cognitive testing was evaluated using the Delayed Word Recall Test, the Digit Symbol Subtest of the Wechsler Adult Intelligence Scale-Revised (WAIS-R) test, and the Controlled Oral Word Association (or Word Fluency) Test of the Multilingual Aphasia Examination [55].

### 4.3. Electrochemiluminescence Immunoassays (ECLIAs)

ECLIAs for measuring IL-1β protein levels were performed using the V-PLEX Human IL-1β Kit (Cat #K151QPD, MSD), as described in [56]. Briefly, reagents were prepared, including calibrator standards. Plates were washed in washing buffer, and 50 μL of samples and calibrators was loaded in each well of the 96-well kit, followed by overnight incubation at 4 °C. Each plate was then washed, and 25 μL of detection antibody solution was added for 2 h at room temperature while shaking, followed by another wash and the addition of 150 L of 2X Read Buffer T. Each plate was then read using MESO-QuickPlex SQ-120 and DISCOVERY WORKBENCH 4.0 software (MSD).

Similarly, the S-PLEX Human IL-1β Kit (Cat #K151ADSS) was used according to manufacturer’s instructions and read using MESO-QuickPlex SQ-120 (MSD). Briefly, the coating and blocking solutions and calibrators were prepared. Plates were first coated and then washed, and then the blocking step was carried out. Then, 25 μL of sample was added to each well and incubated at room temperature for 1.5 h while being shaken at 700 rpm at room temperature for 1.5 h. TURBO-BOOST antibody solution was then prepared and added to each well for incubation at room temperature for 1 h while being shaken at 700 rpm. Then, Enhanced solution was prepared and added to the plate for incubation at room temperature for 30 min while being shaken at 700 rpm. TURBO-TAG Detection Solution was then prepared and added to each well for incubation at 27 °C for 1 h while being shaken at 700 rpm, and then the plates were read.

### 4.4. Single-Molecule Array (Simoa)

IL-1β protein levels were measured using the IL-1β 3.0 kit (Quanterix) according to the manufacturer’s instructions and the method described in [43]. Calibrators and controls were prepared, and RGP 2.0 Reagent bottles were baked at 800 rpm for at least 30 min at 30 °C. Then, 182 μL of calibrators, controls and samples were loaded into the Simoa 96-well plate. Reagents and samples were loaded into the respective bays of the HD-X Analyzer, and then the assay was set up to be run by a computer.

### 4.5. Statistical Analyses

Prism 10.0 software (GraphPad Software, La Jolla, CA, USA) was used for statistical analyses. Outliers were calculated via the ROUT method, as described in [57], followed by calculation of descriptive statistics. Normality was determined using the Shapiro–Wilk test. Statistical comparisons between two groups were made using the Mann–Whitney test since all data were non-parametric. Then, ROCs were calculated to obtain the sensitivity and specificity as well as the likelihood ratios. Multiple group comparisons were made using ANOVA followed by the Kruskal–Wallis test. Correlations between were made using Spearman correlation, and the degree of agreement of detection between methods was assessed using Bland–Altman analysis. The *p*-value of significance used was <0.05, and all outcome measures were evaluated by investigators who were blinded to the experimental groups.

## 5. Conclusions

We have shown that the Simoa IL-1β 3.0 assay provided the greatest sensitivity in distinguishing IL-1β levels in the serum of AD patients and HC subjects when compared to the S-Plex and V-Plex assays. Moreover, S-Plex was superior to V-Plex. Importantly, our data suggest that all three assays were effective in measuring IL-1β levels in affected individuals who had elevated levels of this cytokine. However, Simoa 3.0 demonstrated the greatest sensitivity in measuring lower concentrations of IL-1β in the patients’ serum. The identification of IL-1β as a promising diagnostic and/or theragnostic biomarker of the inflammatory response in AD has the potential to open novel therapeutic avenues for treating AD-related inflammation. Thus, studies are underway to better understand the biomarker role of IL-1β associated with the inflammatory response present in neurodegenerative diseases such as AD and Parkinson’s disease. The current study shines light on some of the most reliable methods available for measuring IL-1β levels in patients’ samples.

## Figures and Tables

**Table 1:** ROC analysis.

Assay	Area	Std. Error	95% C.I.	*p*-Value
V-Plex	0.57	0.13	0.32–0.81	0.59
S-Plex	0.67	0.09	0.49–0.84	0.08
Simoa	0.95	0.04	0.88–1.00	<0.0001

**Table 2:** Cut-off points in serum for AD patients.

Assay	Cut-Off Point (pg/mL)	Sensitivity (%)	Specificity (%)	LR	PPV (%)	NPV (%)	Accuracy (%)
V-Plex	>0.03132	75%	56%	1.688	75%	56%	68%
S-Plex	>0.08862	83%	42%	1.439	60%	71%	6%
Simoa	>0.05378	100%	89%	9.5	91%	100%	95%

## Data Availability

Data are contained within the article. Further data will be provided upon request to the corresponding author.
